# Supplementary material for: Validity of heart rate measurements in wrist-based monitors across skin tones during exercise
Source: PLoS One. 2025 Feb 10;20(2):e0318724. doi: 10.1371/journal.pone.0318724 (PMC11809914; doi:10.1371/journal.pone.0318724)

Supporting Information

Manuscript Title: Validity of heart rate measurements in wrist-based monitors across skin tones during exercise
Manuscript ID: PONE-D-24-32801

Statistical analysis assumptions testing for linear mixed effects model

Model Description: HR error was the dependent variable. Fixed effects were exercise intensity (low, moderate and high), skin tone (light, medium, and dark), and the interaction effects between exercise intensity and skin tone. We included participants as a random effect.

S1 Fig. Homogeneity of variance

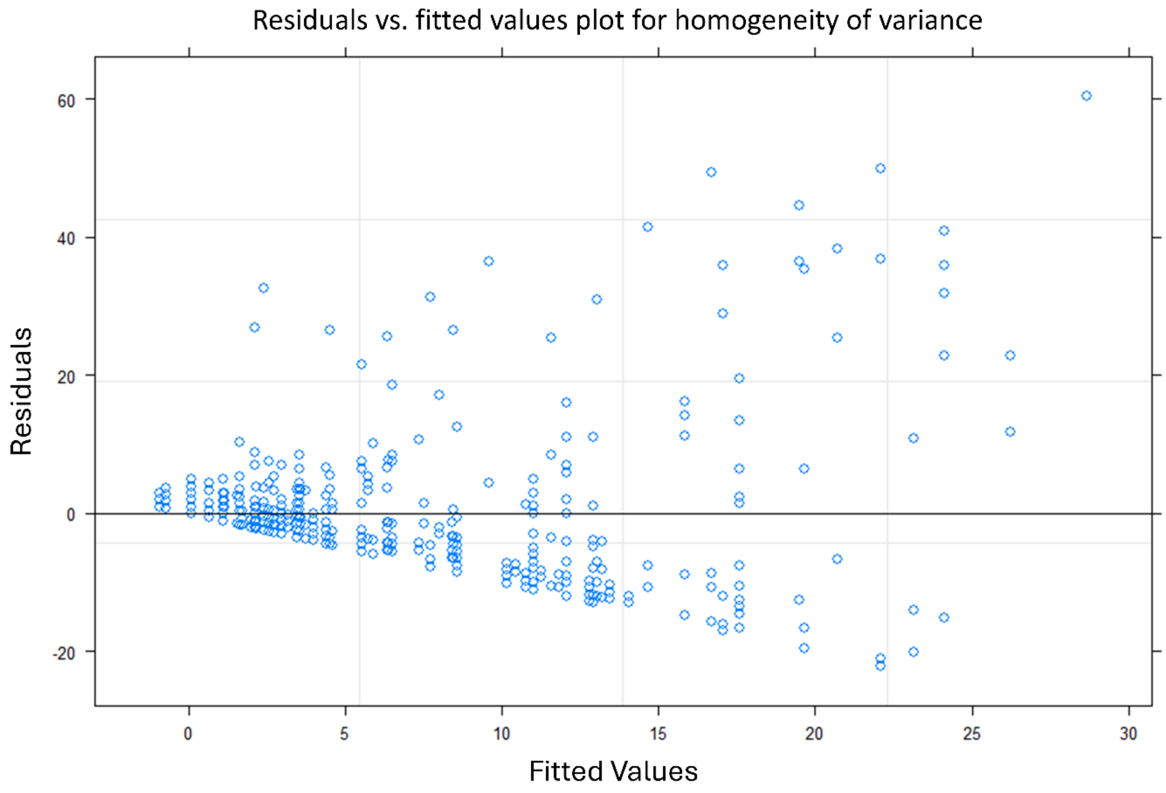

S2 Fig. Histogram of normality of residuals (fixed effects)

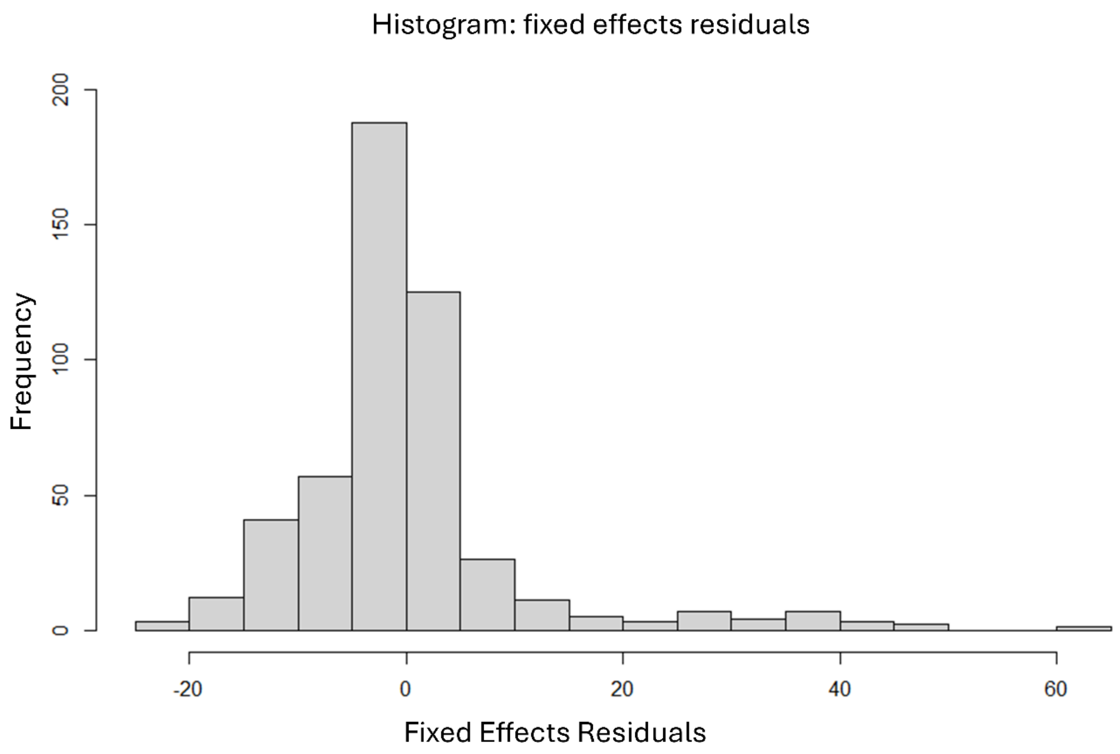

S3 Fig. Q-Q plot of normality of residuals (fixed effects)

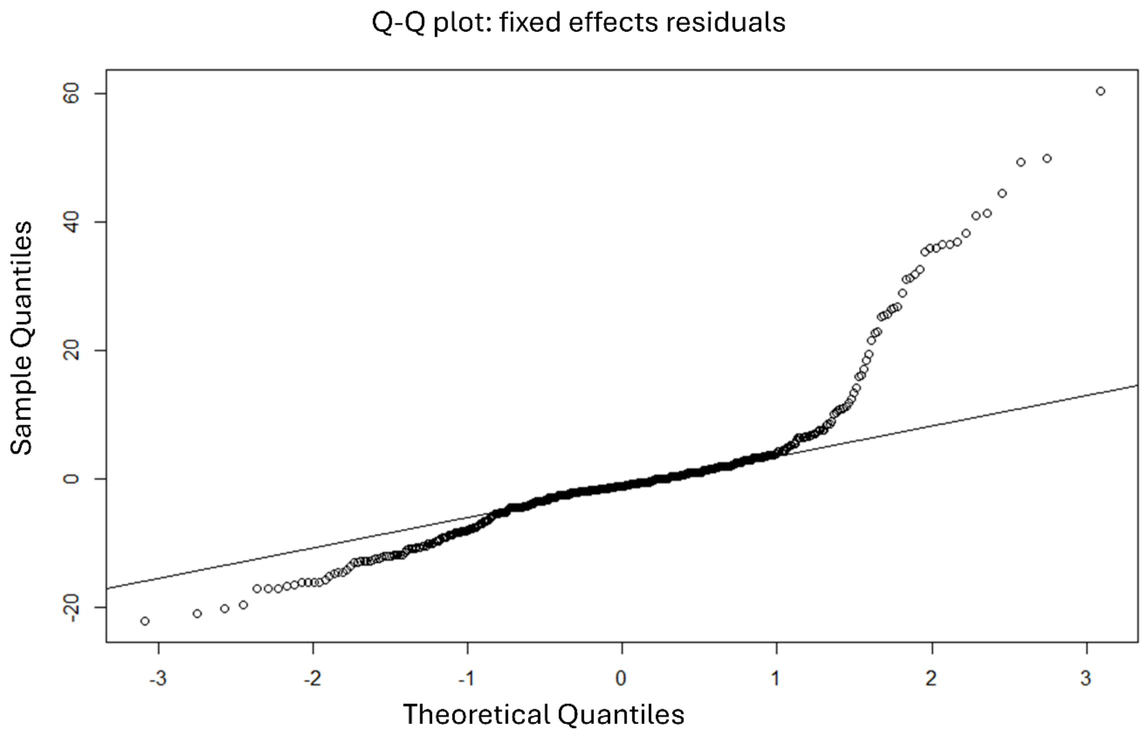

S4 Fig. Histogram of normality of residuals (random effects)

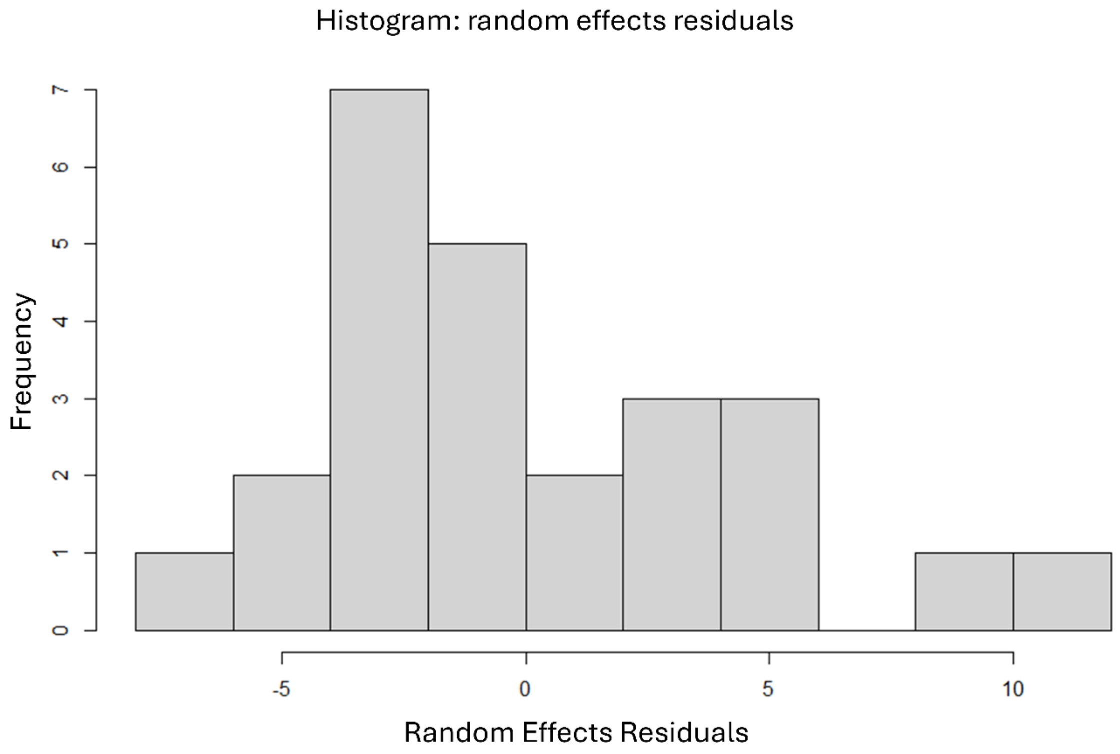

S5 Fig. Q-Q plot of normality of residuals (random effects)

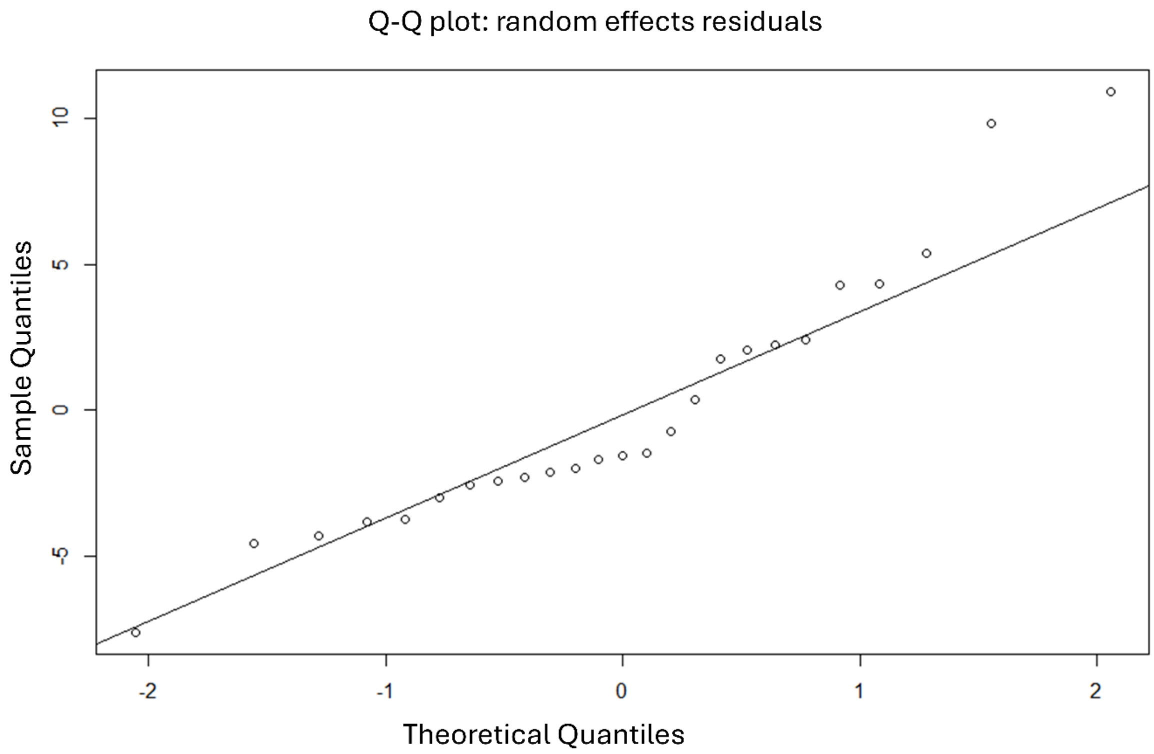

Supplement: S1 File — Results for the model assumptions testing for the linear mixed effects model. (PDF) [file pone.0318724.s001.pdf]
