# Supplementary material for: Validity of heart rate measurements in wrist-based monitors across skin tones during exercise
Source: PLoS One. 2025 Feb 10;20(2):e0318724. doi: 10.1371/journal.pone.0318724 (PMC11809914; doi:10.1371/journal.pone.0318724)
Supplement: Attachment — Submitted filename: Response to Reviewers.docx [file pone.0318724.s002.docx]

October 11, 2024

Manuscript Title: Validity of heart rate measurements in wrist-based monitors across skin tones during exercise

Manuscript ID: PONE-D-24-32801

Dear Dr. Emily Chenette and Reviewers,

Thank you reviewing our manuscript and for the opportunity to respond to the comments for re-submission.

The following content includes a copy of changes requested as per journal requirements and of each reviewer’s comments, followed by our response under each comment. The page and line numbers of the revisions refer to the tracked changes version of the revised manuscript.

Sincerely,

Janice Eng, PT, PhD

Professor

Centre for Aging SMART, Vancouver Coastal Health

4255 Laurel Street, Vancouver BC, V5Z 2G9 Canada

Department of Physical Therapy, Faculty of Medicine

University of British Columbia

**Journal requirements:**

When submitting your revision, we need you to address these additional requirements.

**1. Please ensure that your manuscript meets PLOS ONE's style requirements, including those for file naming.**

Response: We have made specific edits to align with PLOS ONE’s style requirements. This includes all heading levels, and figure and table titles and captions throughout the manuscript.

**2. Please note that PLOS ONE has specific guidelines on code sharing for submissions in which author-generated code underpins the findings in the manuscript. In these cases, we expect all author-generated code to be made available without restrictions upon publication of the work. Please review our guidelines at https://journals.plos.org/plosone/s/materials-and-software-sharing#loc-sharing-code and ensure that your code is shared in a way that follows best practice and facilitates reproducibility and reuse.**

Response: The statistical analysis code for the data analysis is available on the corresponding author’s Borealis (A Canadian Dataverse Repository) account (<https://doi.org/10.5683/SP3/LLD99A>). An updated Data Availability Statement has been included to the submission.

**3. Thank you for stating the following financial disclosure:**

**“Funding for the current study was supported by the Canada Research Chairs Program (to J Eng), Michael Smith Health BC Postdoctoral Award (to S Hung), and the Canadian Institutes of Health Research Foundation Grant (FDN 143340; to J Eng).”**

**Please state what role the funders took in the study. If the funders had no role, please state: "The funders had no role in study design, data collection and analysis, decision to publish, or preparation of the manuscript."**

**If this statement is not correct you must amend it as needed.**

**Please include this amended Role of Funder statement in your cover letter; we will change the online submission form on your behalf.**

Response: The role of the funder has been included in the updated cover letter. Thank you for amending the Role of Funder statement on the online submission form.

**4. Thank you for stating the following in the Acknowledgments Section of your manuscript:**

**“Funding for the current study was supported by the Canada Research Chairs Program (to J Eng), Michael Smith Health BC Postdoctoral Award (to S Hung), and the Canadian Institutes of Health Research Foundation Grant (FDN 143340; to J Eng). The study team would like to thank the study participants and Asahi Ng, Jackson Liu, and Nadine Akbarali for contributing to participant recruitment and data collection.”**

**We note that you have provided funding information that is currently declared in your Funding Statement. However, funding information should not appear in the Acknowledgments section or other areas of your manuscript. We will only publish funding information present in the Funding Statement section of the online submission form.**

**Please remove any funding-related text from the manuscript and let us know how you would like to update your Funding Statement. Currently, your Funding Statement reads as follows:**

**“Funding for the current study was supported by the Canada Research Chairs Program (to J Eng), Michael Smith Health BC Postdoctoral Award (to S Hung), and the Canadian Institutes of Health Research Foundation Grant (FDN 143340; to J Eng).”**

**Please include your amended statements within your cover letter; we will change the online submission form on your behalf.**

Response: We have removed the funding statement from the manuscripts text acknowledgements. No changes to the Funding Statement are required.

**Reviewer's Responses to Questions**

Reviewer #1:

**To enhance the depth of your manuscript, you might consider including references to additional studies that explore related aspects of heart rate monitoring and physiological assessments. These studies could provide valuable context and further support the findings of your research. Including such references might offer readers a more comprehensive understanding of the topic and its broader implications (Sampaio, T., Morais, J. E., & Bragada, J. A. (2024). StepTest4all: Improving the Prediction of Cardiovascular Capacity Assessment in Young Adults. Journal of Functional Morphology and Kinesiology, 9(1), 30. And Bragada, J. A., Magalhães, P. M., São-Pedro, E., Bartolomeu, R. F., & Morais, J. E. (2024). Net Heart Rate for Estimating Oxygen Consumption in Active Adults. Journal of Functional Morphology and Kinesiology, 9(2), 66.).**

Response: Thank you to the reviewer for suggesting these references and associated topics. We included the reviewer’s second suggested reference (Bragada, J. A., Magalhães, P. M., São-Pedro, E., Bartolomeu, R. F., & Morais, J. E. (2024). Net Heart Rate for Estimating Oxygen Consumption in Active Adults. Journal of Functional Morphology and Kinesiology, 9(2), 66.) as it specifically pertains the heart rate measurements in the estimation of oxygen consumption, and highlights the importance of accurate heart rate in monitoring exercise response, exercising testing and estimating oxygen consumption.

Page 3; Line 48: The popularity of wearable fitness trackers to monitor HR has increased in recent years for consumers in the general public, healthcare, and research settings (5), including monitoring HR for exercise prescription or estimating oxygen consumption during exercise testing (6).

2. INTRODUCTION

**Please see this reference about devices’ metrological characteristics: Bouillod, A., Soto-Romero, G., Grappe, F., Bertucci, W., Brunet, E., & Cassirame, J. (2022). Caveats and recommendations to assess the validity and reliability of cycling power meters: A systematic scoping review. Sensors, 22(1), 386.**

Response: Thank you for this suggestion, and for outlining the different properties of validity to note. In the first instance of describing validity, we have clarified that we are referring to accuracy of heart rate measurements.

Page 3; Line 53: For these reasons, wireless, ECG-based chest strap HR monitors have grown in popularity and are valid (i.e., accuracy of measuring heart rate) alternatives to ECG (6).

**(P.11 l.37 to 52 and 53 to 62) – The transition between the discussion on the general importance of HR monitoring during exercise and the specific focus on PPG technology across skin tones is somewhat abrupt. The manuscript would benefit from a smoother transition, perhaps by first addressing the general challenges of HR monitoring technologies before narrowing down to the specific issue of skin tone.**

Response: We added general context of challenges with PPG technology for heart rate measurement prior to discussing skin tone.

Page 3; line 66: Accurate measurements of HR using PPG devices is highly dependent on PPG signal accuracy, which can be influenced by multiple factors, including type of sensors used (example, shape of sensors and light intensity), post-processing algorithms used, the deformation of the skin when device is worn, vascular aging (example, arterial stiffness), and excessive movement (15).

**(P.11 l.53 to 62) – While the impact of skin tone on PPG accuracy is mentioned, there is insufficient depth in the review of existing literature. The paragraph should include references to key studies that have previously explored this issue, offering a more comprehensive context for the reader.**

Response: In our initial submission, we included a key reference to the systematic review completed by Koerber and colleagues, who used rigorous systematic review methods to investigate the impact of skin tone on accuracy of heart rate measures using wrist-based devices. To provide more comprehensive context to the reader, we provided more details on interpretation of the existing literature to date by Koerber and colleagues, such as elaborating on the potential reasons for inconsistent results within the existing literature (inadequate sample size, non-standardized use of skin tone categorization) and how our study adds to the literature.

Page 4; line 76: Another systematic review (10 studies, 469 participants, 26 different devices) rigorously examined the available literature on the impact of skin tone on the accuracy of HR measurements using wrist-based PPG devices (18). The authors concluded that there are conflicting results in HR measurement accuracy across different skin tones, with four studies observing lower accuracy with darker skin tones, four observing no difference, and two having mixed results (18). The authors attributed the mixed results partly due to inadequate sample sizes, use of multiple devices for comparison, and inconsistent methods with categorizing skin tones (example, non-standardized self-reported race and ethnicity) (18).

**(P.12 l.60 to 62) – The issue of skin tone affecting PPG accuracy is mentioned, but the introduction does not sufficiently explain why this is a significant problem. Providing context, such as discussing the implications for diverse populations or the potential for health disparities, would make the rationale for the study clearer.**

Response: We provided more context by introducing the issue with HR inaccuracies when monitoring exercise for clinical population

Page 5; line 94: This potential source of inaccuracy could have important implications for use across ethnically-diverse populations, especially when HR inaccuracies could pose a safety risk, particularly in clinical settings for people with darker skin (18,24). In clinical settings, such as people with cardiovascular diseases, monitoring HR during exercise is recommended to ensure people are safely exercising at HR below the onset of ischemic symptoms and at an adequate intensity to maximize exercise adaptations and benefits (31). People with darker skin tones (example, Black and Hispanic ethnic backgrounds) are at higher risk of cardiovascular diseases (32), and inaccurate HR measurements using PPG-based devices would result in potential health disparities with exercise safety and treatment efficacy in this clinical population.

METHODS

**(P.13 l.86 to 101) – The subsection titled "Study Procedures" provides a general overview of the experimental protocol. However, the clarity and comprehensibility of this section would be greatly enhanced by dividing the content into more specific subsections.**

Response:

Page 7; 143 and Page 8; line 158::“Skin tone categorization” and “Resting and exercise testing heart rate” subsections have been added.

**(P.14 l.118) – It is suggested that the authors present examples of each skin tone category used in the study. This could include representative images or a clear description of how skin tones were classified.**

Response: We included a description of the skin tone categories in table form (Table 1; all tables number adjusted thereafter).

Page 7; line 146: …the Fitzpatrick Scale, which categorizes skin tones across 6 types based on colour and ultraviolet radiation response (36). Table 1 describes the 6 Fitzpatrick Scale skin tone categories (37).

Table 1 has been added to Page 7; line 156.

**(P.15 l.145) – The choice of a linear mixed-effects model is appropriate given the study's design. However, the manuscript does not discuss the assumptions underlying this model or whether these assumptions were tested (e.g., normality of residuals, homoscedasticity).**

Response: We thank the reviewer for the opportunity to expand on the appropriateness of linear mixed effects models in the current study. We tested the assumptions of our model (results found in Supporting Information File 1, S1_File.pdf), and observed mild heteroskedasticity of variance and mild positive skewness in our fixed effects residuals. Linear mixed effects models have been shown to be robust to such violations of distributional assumptions. We included three key references in support of the robustness of the linear mixed effects models:

45.	Harrison XA, Donaldson L, Correa-Cano ME, Evans J, Fisher DN, Goodwin CE, et al. A brief introduction to mixed effects modelling and multi-model inference in ecology. PeerJ. 2018 May 23;6:e4794. (

46.	Schielzeth H, Dingemanse NJ, Nakagawa S, Westneat DF, Allegue H, Teplitsky C, et al. Robustness of linear mixed-effects models to violations of distributional assumptions. Methods Ecol Evol. 2020;11(9):1141–52.

47.	Jacqmin-Gadda H, Sibillot S, Proust C, Molina JM, Thiébaut R. Robustness of the linear mixed model to misspecified error distribution. Comput Stat Data Anal. 2007 Jun 15;51(10):5142–54.

We updated the manuscript in the Statistical Analysis to include the results of assumption testing for our linear mixed effects model:

Page 10; line 203: We tested the linear mixed effects model assumptions (see S1 File for assumptions results). We observed mild heteroskedasticity of variance (S1 Fig in S1 File) and mild positive skewness in our fixed effects residual distribution (S2 Fig and S3 Fig in S1 File). Linear mixed effects models are robust to violation to distributional assumptions and heteroscedasticity and remains an appropriate model for our study data (45–47).

Page 28; line 538: We added the Supporting Information List at the end of the manuscript.

3. RESULTS

**(P.17 l.174) - The authors provide detailed information about the sample recruitment process and the characteristics of the participants in the Results section. While this information is crucial, it would be more appropriately placed in the Methods section.**

Response: We thank the reviewer for the suggestion. We have outlined procedures we followed to recruit study participants in the Methods section. We believe that the outcome of the participant recruitment procedures we followed, including the sample recruitment process (number of people who expressed interested, were screened and recruited, and later excluded), and characteristics of the participants, is better suited in the Results section.

**(P.17 l.174) – Additionally, it is suggested that the authors include a graphical representation of the sample recruitment process, such as a flowchart or diagram.**

Response: A recruitment flow chart has been included, and is now labelled as Fig1 (all figure numbering has been updated).

Figure caption has been added to Page 11; line 236: Fig1. Study recruitment process flowchart.

**(P.17 l.188) – The characteristics of the sample, currently presented in the Results section, should also be reallocated to the Methods section.**

Response: We thank the reviewer for the suggestion. We believe that the outcome of the participant recruitment procedures, including the characteristics of the participants, is better suited in the Results section.

**(P.17 l.188) – Regarding Table 1, the presentation of "mean and SD" (standard deviation) should be adjusted. Currently, it appears in the line of the variable; however, it would be clearer and more conventional to include "mean ± SD" in the column header where the data is presented.**

Response: In all tables, we have changed all presentations of “mean (SD)” to “mean ± SD” to align with convention, where applicable. We did not include this in the column header for this table (now Table 2), given that the “Female” variable is not presented in means and standard deviations.

**(P.17 l.188) – Additionally, the meaning of "SD" should be clarified in a footnote to the table.**

Response: This has been clarified in the footnotes of all tables.

**(P.22 l.241) – Provide Figure 1 with more quality.**

Response: A higher quality Bland-Altman plot (now Fig2) has been included.

4. DISCUSSION

**(P.23 l.261) – Add a period after the reference and check for similar instances throughout the manuscript.**

Response: We have corrected these were applicable.

5. REFERENCES

**Verify that all references are formatted according to the journal guidelines.**

Response: We reviewed the reference list and corrected the formatting where applicable, particularly the journal abbreviations.

Reviewer #2:

**The authors should provide a definition of heart rate reserve.**

Response: We defined heart rate reserve as the maximum heart rate minus the resting heart rate in the Methods section of the study. We also took to opportunity to correct the references for equations to calculate heart rate reserve.

Page 9; line 177: Maximum HR was estimated using the Karvonen equation [HRmax = 206.9 - (0.67 x age)] (41) (36), and used with resting HR to calculate HR reserve (HRR, Maximum HR minus resting HR) (42). Each study visit lasted approximately 45 minutes.

**Referring to line 82, the fact that the authors spoke of a “primary aim” implies that there is at least a secondary one: what is it? Or do the authors mean something different? I encourage the authors to clearly express the aim/s of their study.**

Response: We added that the secondary aim and hypothesis are the end of the Introduction.

Page 5; line 109: The secondary aim of this study was to evaluate the accuracy of the wrist-based HR monitors at rest across different skin tones. We hypothesize that accuracy of measurements will not differ between different skin tones.

**How were the HR measurements from the two devices synchronized to be considered time-matched?**

Response: Under the Study Procedures section in the Methods, we clarified how the two devices were synchronized.

Page 9; line 175: To ensure HR readings from the POL and FB5 devices were recorded at the same time, one investigator was assigned to record HR data from each device and a central timer was used to ensure readings were recorded at the same time. A verbal countdown facilitated the two devices being started at the same time (within a second)

**At line 196 the measurement unit is missing. I encourage the authors to always express it.**

Response:

Page 12; line 246: Beats per minute was added as “bpm”.

**An aspect should be clarified at line 126:** **the authors stated that they took the lowest HR observed after five minutes of quiet sitting as resting HR. If they took the minimum HR, they considered a time interval of observation after (then subsequently) 5 minutes of quite sitting: what is the time duration of this interval?**

Response: Thank you for the opportunity to clarify this point. The heart rate observe at the end of 5 minutes was taken. This was corrected.

Page 8; line 159: A single resting HR measurement was collected from both devices by taking the HR observed at the end of five minutes of quiet sitting.

**I suggest not to take for granted concepts and meaning of parameters (for example, alpha).**

Response:

Page 9: We defined alpha as level of significance (line 184), and power as probability of correctly rejecting the null hypothesis (line 201).

**Explaining the secondary analysis, the authors stated that they “examined the accuracy in resting HR measurements across different skin tones”, and this seems in contradiction with lines 168-170.**

Response: We moved lines 168-170 (now Page 10; 210-212) to a new paragraph after the primary analysis (Page 10; line 209-211) to differentiate from the secondary analysis.

**Tables can be better described in their content and readers should be better guided in their interpretation.**

Response: For Table 3 and Table 4, we re-referenced the results sentences back to the respective tables to improve interpretation

Page 12; line 244: For resting measures, the mean error between the POL and FB5 for all participants was 2.8 bpm (SD: 2.6) and were similar across all skin tone groups (Table 3). During exercise, the mean error remained relatively stable across all exercise intensities for light skin tone (approximately 4 bpm) (Table 4). We reviewed the remaining result tables (Table 4 and 5) and their referenced text in the Results section, and believe it guides the interpretation for readers appropriately.

**In the Bland-Altman plots the indication of the x axis is missing.**

Response: We amended the Bland-Altman plots to include an x-axis (now Fig 2) and uploaded a new version.

**In the discussion, the authors stated that “the small study sample size may have increased the possibility of Type 1 Errors”. Can the authors better explain why? What about Type 2 Errors?**

Response: Thank you for the opportunity to correct and expand on this. The small sample size increases possibility for Type 2 Error for detecting potential significant differences in moderate intensity heart rate between light and medium skins tones.

Page 20; line 359: The small study sample size may have increased the possibility of Type 2 Errors for detecting potential significant differences in moderate intensity (40-60% HRR) between light and medium skin tone.
