# Supplementary material for: Validity of heart rate measurements in wrist-based monitors across skin tones during exercise
Source: PLoS One. 2025 Feb 10;20(2):e0318724. doi: 10.1371/journal.pone.0318724 (PMC11809914; doi:10.1371/journal.pone.0318724)
Supplement: Attachment — Submitted filename: Response to Reviewer Comments.docx [file pone.0318724.s003.docx]

November 14, 2024

Manuscript Title: Validity of heart rate measurements in wrist-based monitors across skin tones during exercise

Manuscript ID: PONE-D-24-32801

Dear Dr. Emily Chenette and Reviewers,

Thank you reviewing our manuscript and for the opportunity to respond to the comments for re-submission.

The following content includes a copy of changes requested as per journal requirements and of each reviewer’s comments, followed by our response under each comment. The page and line numbers of the revisions refer to the tracked changes version of the revised manuscript.

Sincerely,

Janice Eng, PT, PhD

Professor

Centre for Aging SMART, Vancouver Coastal Health

4255 Laurel Street, Vancouver BC, V5Z 2G9 Canada

Department of Physical Therapy, Faculty of Medicine

University of British Columbia

**Journal Requirements:**

**Please review your reference list to ensure that it is complete and correct. If you have cited papers that have been retracted, please include the rationale for doing so in the manuscript text, or remove these references and replace them with relevant current references. Any changes to the reference list should be mentioned in the rebuttal letter that accompanies your revised manuscript. If you need to cite a retracted article, indicate the article’s retracted status in the References list and also include a citation and full reference for the retraction notice.**

**Reviewer #1: I congratulate the authors for improving the manuscript as advised. In my opinion, this can be published in the present form.**

Response: We thank reviewer 1 for their helpful feedback to improve the manuscript.

**Reviewer #2:**

**Can the authors better explain the difference between “alpha” and “power”?**

Response: We further explained the concepts of alpha and power in the statistical analysis to help distinguish the two concepts:

Page 9; line 180: 0.05 alpha (level of significance; 5% probability of incorrectly rejecting the null hypothesis or falsely detecting an effect when there is no true effect [Type 1 Error]).

Page 10; Line 199: 80% power (probability of correctly rejecting the null hypothesis; 80% chance of detecting statistical significance when a true effect is present)

**The authors re-referenced the results sentences back to the respective tables to improve interpretation, but I suggest checking table numbers.**

Response: We corrected references to Table 3 and Table 4 in the results (Page 12; line 241 and 242).

**The current Table 6 should be further described. What is the role of the intercept? What can we deduce from its value? And what about random effects? “ICC” is not even defined (as well as the other random effects). My suggestion is not to take concepts for granted by leaving too much to the reader's interpretation, even for “standard” concepts or parameters. Every content in the table should at least be defined/described. What is shown in the table? Even the acronym “HR” is fairly standard, especially for certain areas of research, but the authors have rightly defined it in Abstract (and should also define it when first used in the text)**

Response: Thank you for this suggestion to clarify the contents and concepts of Table 6. We have clarified interpretation of the intercept, random effects, and intraclass correlation coefficient (ICC). We included these details in the table footer.

Page 17; Lines 284 to 289: Note: the intercept indicates the absolute error in heart rate without including effects of other skin tones (i.e., the effect of light skin tone only). For random effects: an σ2 value of 114.19 bpm indicates a 10.69 bpm (square root of 114.19) of absolute error explained by within-participant variance; a τ00 participants value of 26.10 bpm indicates 5.11 bpm (square root of 26.10) between-participant variance; a 0.19 intraclass correlation coefficient indicates that 19% of the total variance of absolute error of heart rate is attributed to between-participant variance.

We have also clarified that our absolute errors are for heart rate measures in the title and/or appliable column heading for Table 6 (Page 16), along with Table 3 (page 13), Table 4 (Page 13), and Table 5 (Page 14).

HRR has already been defined as heart rate reserve in the table footer.

**In the current figure 2, I suggest specifying that on x axis means are reported and on y axis differences are reported.**

Response: We amended the axis x axis labels to “Mean of Polar H10 and Fitbit Charge 5” and y axis labels to “Difference between Polar H10 - Fitbit Charge 5”
